# Mortality of Sacramento City, Cal., for 1859

**Published:** 1860-04

**Authors:** 


*Mortality of Sacramento City, Cal., for* 1859.—We have been quite interested in the necrological report for the above city, for 1859, by Thos. M. Logan, M. D.

The total mortality was 307. The population of the city the Dr. estimates at 20,000. If this calculation is correct, the deaths are 1 to every 65 of the population, or as 15 to every thousand living, and presents a highly favorable condition of the health of Sacramento.

Pulmonary consumption, according to these statistics is quite fatal, swelling the mortality in 1859, over one-fifth of all the deaths. They numbered 63, or 20.52 per cent.

Dr. Logan is not willing to attribute the prevalence of this disease in California altogether to the immigration of consumptive invalids from the eastern states, as has been heretofore assigned for its ravages. But as we understand him, he takes a more rational view of the cause, and suggests that it is the "result of long continued agencies, springing from extraordinary mental excitement, gross irregularities of life and overtasking of the physical energies, which, by deranging the healthy equilibrium, or insidiously depressing the vital standard, become in systems already pre-disposed to strumous affections the exciting causes, in connection with the peculiar climate of California, which the doctor believes is unfavorable for consumptives. He alludes particularly to the sudden vicissitudes of the atmosphere during the summer nights.

Cholera Infantum, supplies but one death in the year, while convulsions furnish only six. A similar limited proportion when compared with the infant mortality in our Atlantic cities, prevails as to the mortality from other diseases to which children are subject. The number of deaths in children under ten years, was 110 or 35 per cent. of the total mortality from all ages. This calculation does not include the still-born, which amounted to 22.

Diphtheria and croup, which are recorded together under one head, gave 14 deaths. This connexion is made, in order to arrive at a more correct data for the deaths from diphtheria, as Dr. Logan thinks, that many of these cases have been designated in the certificates of deaths by other names, and because the deaths from croup and all of a diphtheritic character, hence he says, "by taking a medium course and assuming that only one-half of all those deaths, which should have been credited to diphtheria, have been registered to other names." He arrives at the true state of its mortality.

While we agree with Dr. Logan, that the confusion of terms which now exists in regard to this formidable disease, (and we would say as much in regard to the loose and confused manner of certifying to deaths, from many other diseases,) is unfortunate, for the exact science of vital statistics, we must dissent from him in his views of croup, that, unless the inflammation becomes so violent as to form a false membrane, it is not croup, but a "simple form of catarrhal affection," and that the "hoarse or croup like sounds, are only harmless symptoms of another disorder." Nor do we believe that because "children are subject to it, (croup,)" hence "it is of an inocuous character." But we cannot follow him up in all his peculiar notions of croup, and would in a word, caution the younger branches of the profession from falling into the error, that when called in the night, to a child laboring under the paroxysm of croup, it "will soon pass off if left to its own course," for the doctor teaches, that any "special treatment is questionable," unless this paroxysm runs into "the membranous disease which it never does."

Croup, according to Dr. Wood, one of our best standard authorities, is "a disease in which inflammation, or high vascular irritation of the laryngeal, or laryngo tracheal mucous membrane, is combined with spasm of the interior of the larynx, giving rise to peculiar modifications of voice, cough and respiration," and while the same able writer admits a catarrhal and pseudo-membranous croup, he says impressively, "no rule in medicine is more certain, than that every case of croup, whatever may be its apparent character, should be treated promptly and efficiently." This is the invariable experience of every sound practioner of medicine, and should never be lost sight of, under any circumstances. —*Medical & Surgical Reporter.*

